# Dynamin2 Organizes Lamellipodial Actin Networks to Orchestrate Lamellar Actomyosin

**DOI:** 10.1371/journal.pone.0094330

**Published:** 2014-04-07

**Authors:** Manisha Menon, Olga L. Askinazi, Dorothy A. Schafer

**Affiliations:** 1 Department of Biology, University of Virginia, Charlottesville, Virginia, United States of America; 2 Department of Cell Biology, University of Virginia, Charlottesville, Virginia, United States of America; Vanderbilt University Medical Center, United States of America

## Abstract

Actin networks in migrating cells exist as several interdependent structures: sheet-like networks of branched actin filaments in lamellipodia; arrays of bundled actin filaments co-assembled with myosin II in lamellae; and actin filaments that engage focal adhesions. How these dynamic networks are integrated and coordinated to maintain a coherent actin cytoskeleton in migrating cells is not known. We show that the large GTPase dynamin2 is enriched in the distal lamellipod where it regulates lamellipodial actin networks as they form and flow in U2-OS cells. Within lamellipodia, dynamin2 regulated the spatiotemporal distributions of α-actinin and cortactin, two actin-binding proteins that specify actin network architecture. Dynamin2's action on lamellipodial F-actin influenced the formation and retrograde flow of lamellar actomyosin via direct and indirect interactions with actin filaments and a finely tuned GTP hydrolysis activity. Expression in dynamin2-depleted cells of a mutant dynamin2 protein that restores endocytic activity, but not activities that remodel actin filaments, demonstrated that actin filament remodeling by dynamin2 did not depend of its functions in endocytosis. Thus, dynamin2 acts within lamellipodia to organize actin filaments and regulate assembly and flow of lamellar actomyosin. We hypothesize that through its actions on lamellipodial F-actin, dynamin2 generates F-actin structures that give rise to lamellar actomyosin and for efficient coupling of F-actin at focal adhesions. In this way, dynamin2 orchestrates the global actin cytoskeleton.

## Introduction

Cells assemble a spectrum of dynamic actin networks comprised of branched, crosslinked and bundled filaments that, together, move cells and organelles, enable cell division and organize cells within tissues. Well-characterized actin networks include the sheet-like, dendritic networks of lamellipodia that support protrusion at cell membranes and the contractile actomyosin networks of lamellae. Interactions between these distinct actin networks occur, in part, at focal adhesions. Much is known about the biochemical mechanisms by which individual actin networks form, but the processes by which different actin networks are integrated in time and space to maintain a dynamic, but coherent, actin cytoskeleton are less well understood.

In migrating cells, lamellipodial, lamellar and focal adhesion-associated actin networks are interdependent. Lamellar actomyosin evolves, in part, from Arp2/3-dependent actin filaments and from remnants of lamellipodial F-actin-rich structures such as filopodia or F-actin of retracting protrusions [1]–[6]. The centripetal flow of lamellar actomyosin is regulated, in part, by the strength of the coupling between the lamellar networks and nascent adhesions [6]–[8]. Similarly, interactions of lamellipodial F-actin at nascent adhesions influence lamellipodial actin network organization and dynamics [9]. Adhesions initiated within lamellipodia mature in response to forces generated by lamellar actomyosin [10]. How lamellipodial actin filaments transition from their characteristic dendritic organization to engage nascent focal adhesions or generate actomyosin of the lamellum is not known. Although the molecular mechanisms that regulate the interdependent spatial organization of actin filament networks near the leading edge are likely complex, we report here that the large GTPase dynamin2 is involved.

Dynamin2 is increasingly recognized as a regulator of the actin and microtubule cytoskeletons, but its mechanisms of action and the functions executed by dynamin2 on cytoskeletal filaments are unknown [11]–[16]. Dynamin2 localizes with dendritic, Arp2/3-dependent actin networks in several types of cells and also associates with several actin regulatory proteins (reviewed in [17]). Dynamins also directly bind and bundle actin filaments *in vitro* [18], an activity that is enhanced by cortactin that binds both dynamin's C-terminal proline-rich domain (PRD) and actin filaments [19]–[21]. In cultured cells, dynamin2 influenced the actin cytoskeleton in a cell-type and context-specific manner as determined primarily from dynamin2-depletion experiments. In osteosarcoma cells, dynamin2 was implicated in organizing lamellar actomyosin [21]. Similarly, in epithelial cells, dynamin2 depletion perturbed cell-cell junctions and expressed mutant dynamin2 increased contractility of apical actomyosin [20]. In podocytes from kidney glomerulus, dynamin2 maintained actin stress fibers that anchored podocytes to the culture substratum [18]. In fibroblasts genetically depleted of dynamin1 and dynamin2, F-actin accumulated with clathrin-coated structures at the ventral cell surface [22]. Finally, dynamin organized F-actin of podosomes and invadosomes and maintained invasive activity by v-Src-transformed fibroblasts [23]. Most studies of dynamin's actin-related functions were carried out using fixed cells, making it difficult to deduce mechanisms by which dynamin2 acts on actin networks. In this study, we investigated dynamin functions on lamellipodial and lamellar actin networks in living U2-OS cells. We identify a role for dynamin2 in remodeling lamellipodial actin networks that influences lamellar actomyosin assembly and flow.

## Materials and Methods

### Plasmids and antibodies

Plasmids to express WT or mutant fluorescent protein (FP)-tagged rat dynamin2aa (dyn2) were constructed from the cDNA of rat dynamin2aa (a gift from Mark McNiven, Mayo Clinic, Rochester, MN); cDNAs were subcloned into the EcoR1/HindIII sites of pmCherry-C1 (pmCh-C1) or pEGFP-C1 (Clontech, Mountain View, CA, USA) using standard procedures. Site-directed mutagenesis was accomplished using the QuickChange II kit (Agilent Technologies, Santa Clara, CA, USA) and primers from Integrated DNA Technologies, Inc. (Coralville, IA, USA) or Eurofins MWG Operon (Huntsville, AL, USA). Three rounds of mutagenesis reactions were performed to prepare dyn2-K_5_E_5_, introducing K-to-E mutations at positions 414, 415, 419, 421 and 426 (numbers correspond to rat dyn2). GFP-WT-dyn2 was a gift from Dr. K. Nakayama [24]. Plasmids to express GFP-actin, GFP-α-actinin, mCh-α-actinin and GFP-MLC2 were from Dr. Rick Horwitz (University of Virginia, Charlottesville, VA), GFP-paxillin was from Dr. Martin Schwartz (Yale School of Medicine, New Haven, CT), RFP-cortactin was from Dr. James Casanova (University of Virginia, Charlottesville, VA) and mCh-dynamin1 was from Dr. David Castle (University of Virginia, Charlottesville, VA). Primary antibodies used in this study are listed in Table S1. Secondary antibodies were from Jackson ImmunoResearch (West Grove, PA, USA), LI-COR Biosciences (Lincoln, Nebraska, USA) and Invitrogen (Grand Island, NY, USA).

### Cell culture and transfections

U2-OS cells (ATCC HTB-96, Manassas, VA, USA) were grown in Dulbecco's modified Eagle's medium (DMEM) supplemented with 10% fetal bovine serum (FBS), glutamine, sodium pyruvate and non-essential amino acids. Dynamin2 was depleted using siGenome siRNAs (Dharmacon, Lafayette, CO, USA) targeting human dynamin2 (D2-18, AGUCCUACAUCAACACGAA (catalog number D-004007-18)); a non-targeting siRNA was the control siRNA (catalog number D-001210-05). siRNAs (2 μg) were delivered into cells using an Amaxa Nucleofector II, program X-001, and nucleofection kit V (Amaxa Biosystems, Lonza, Basel, Switzerland) according to the manufacturer's protocol. Cells were used 48 h after nucleofection. Cells were transfected with plasmids (1–1.2 μg) using FuGene HD (Promega, Fitchburg, WI, USA) as directed by the manufacturer and cultured ∼16 hours before imaging.

### Live-cell imaging

U2-OS cells were plated for 3–4 hours on glass-bottomed dishes (MatTek Corporation, Ashland, MA, USA) coated with 2 μg/ml fibronectin (Sigma, St. Louis, MO, USA); 30 min prior to imaging, media was replaced with ‘movie media’ (phenol-red free MEM supplemented with 10% FBS, glutamine and 20 mM HEPES, pH 7). Confocal microscopy was performed using a Nakagawa spinning disc and inverted microscope (Zeiss, Jena, Germany), 63X, 1.4 n.a. objective lens, lasers to excite GFP (488 nm) and mCherry (587 nm), dual 512×512 EM X2-CCD cameras or an ORCA R2 (1344×1024 pixel) camera (Hamamatsu, Bridgewater, NJ, USA), all controlled by Metamorph software. Cells were maintained in a humidified chamber at 37°C during microscopy. A Definite Focus controller (Carl Zeiss, Jena, Germany) was used to maintain focus during collection at a single focal plane.

Total internal reflection fluorescence microscopy was performed on an Olympus X71 inverted microscope equipped with a 60x, 1.45 n.a. oil objective lens, an Argon laser and an ORCA-Flash 4.0 CMOS camera (Hamamatsu). Images were collected at 2 s/frame over 5–10 min as controlled using Metamorph software.

### Immunofluorescence localization

For immunofluorescence detection, cells were fixed for 10 min at 37°C with 3% paraformaldehyde in IF buffer (127 mM NaCl, 5 mM KCl, 1.1 mM NaH_2_PO_4_, 0.4 mM KH_2_PO_4_, 2 mM MgCl_2_, 5.5 mM glucose, 1 mM EGTA and 20 mM Pipes, pH 7.3), permeabilized 10 min in IF buffer containing 0.1% (vol/vol) Triton X-100, blocked and incubated with antibodies and/or 7.5 nM rhodamine-phalloidin. Coverslips were mounted in 1% n-propylgallate in 50 mM TrisCl, pH 8.5, containing 50% glycerol. Micrographs were collected using a Zeiss LSM510 confocal microscope equipped with a 63X, 1.4 n.a. objective lens; images were collected at a focal plane or multiple focal planes spaced 0.5 μm apart. Images composed of multiple focal planes were rendered as a single maximum-intensity, projected image using ImageJ. All images were processed for final figures using Adobe Photoshop.

### Quantitation of receptor internalization

Internalization of transferrin receptor (TfnR) or β1-integrin was quantified using flow cytometry with slight modifications as described [25]. Cells (8×10^4^ cells/well in a 12-well plate) were washed with ice-cold HyQCCM1 media containing 0.2% BSA and 0.01 M HEPES, pH 7.0 (HyQ) and incubated on an ice-slurry 1 h with either PE-conjugated mouse anti-integrin β1 or PE-conjugated mouse anti-TfnR diluted in HyQ. After three washes in ice-cold HyQ, warm HyQ was added and cells incubated at 37°C for varying periods of time. At each time point, warm media was replaced with ice-cold HyQ and incubated in ice for 30 min with AlexaFluor-647 donkey anti-mouse to label surface-associated anti-receptor antibody. After washing in HyQ, cells were detached using Accutase (Innovative Cell technologies, San Diego, CA, USA) and analyzed by flow cytometry (FACSCalibur, Becton Dickinson, Franklin Lakes, NJ, USA). The fluorescence intensities of PE-conjugated anti-receptor antibody (which represents the total surface-associated pool before internalization) and of AlexaFluor647-conjugated antibody (which binds receptor-PE-antibody complex remaining on the surface) at each time point were normalized to the respective fluorescence intensity at zero time to determine relative amounts of total receptor and surface-bound receptor. Antibody remaining on the surface after internalization was obtained from the ratio of normalized AlexaFluor647-labeled antibody fluorescence/normalized PE-conjugated antibody fluorescence for each time point; the inverse of this value represents the percentage of internalized receptor.

### Quantitative western blotting

Whole cell lysates were prepared from 2×10^5^ cells suspended in 200 μl hot 2XSDS sample buffer and heated at 95°C. To quantify phosphorylated proteins, cell lysates were prepared from 4×10^5^ cells plated overnight on 60 mm dishes; cells were washed with ice-cold PBS and lysed by scraping into 250 μl hot 1X SDS sample buffer containing Roche protease inhibitor cocktail, 0.2 mM PMSF and 2 mM sodium orthovanadate. Whole cell lysates were subjected to electrophoresis [26] followed by transfer to nitrocellulose for immunoblotting. Blots were blocked in 5% milk or LiCOR blocking buffer (for phospho-proteins) (Lincoln, Nebraska, USA), incubated with primary antibodies and IR-dye-conjugated secondary antibodies and quantitatively analyzed using a LiCOR Odyssey infrared imager (Lincoln, Nebraska, USA).

### Image analysis

Lamellar retrograde flow was determined from movies of cells expressing GFP-MLC2 collected at 10 s/frame over 10 min using an EM-CCD camera. Kymographs were generated from pixel-wide lines drawn orthogonal to the cell edge at regions where transverse arcs formed using the kymography plugin available in ImageJ (National Institute of Health, Bethesda, MD, USA). The rate of lamellar retrograde flow was determined from the slope of lines in the kymographs that represent individual segments of lamellar actomyosin arcs. Data were exported to MS Excel and GraphPad Prism for analysis and graphing.

The spatiotemporal analysis of GFP-α-actinin within lamellipodia was determined from kymographs of timelapse movies of cells expressing GFP-α-actinin collected at 5 s/frame over 10 min using an ORCA R2 camera. Kymographs were generated from pixel-wide lines drawn orthogonal to the cell edge at regions where lamellipodia were actively protruding. The lamellipodial region in each kymograph was selected as a region-of-interest (ROI) and the standard deviation and the mean value of the GFP-α-actinin fluorescence intensity within the ROI was determined using the Measure function in ImageJ. The standard deviation in GFP-α-actinin fluorescence/mean GFP-α-actinin fluorescence within the ROI (SD/mean) was defined as a measure of the spatiotemporal distribution of GFP-α-actinin within the lamellipod.

The spatial distribution of cortactin was determined from the fluorescence intensity distribution of RFP-cortactin in living cells imaged at 3 s/frame over 7 min. Background was subtracted and the fluorescence intensity along 8-pixel wide line scans drawn orthogonal to protruding regions of the leading edge was recorded; data were normalized to the maximal RFP-cortactin intensity along the line scan, which occurred at the edge of the lamellipod. Scans from multiple kymographs were aligned based on the position of maximal RFP-cortactin fluorescence intensity.

Focal adhesions were analyzed in movies of cells expressing GFP-paxillin and mCh-α-actinin, plated for 2–3 hours on 2 μg/ml fibronectin and collected at 10 s/frame for 20 min at a focal plane corresponding to the cell-substrate interface using dual EM X2-CCD cameras. The length and width of newly formed adhesions were determined from frames of the GFP-paxillin movies using measurement tools in ImageJ. The rate of assembly of GFP-paxillin at nascent adhesions was quantified from the change in GFP-paxillin fluorescence intensity vs. time at ROIs where new adhesions formed; data were plotted as ln(I_t_)/(I_0_) vs. time, where I_t_ is intensity at each timepoint and I_0_ is the initial intensity, as described [27]. Similarly, the rate of accumulation of mCh-α-actinin at adhesions was determined from graphs of ln(I_t_)/(I_0_) vs. time. Background fluorescence obtained from ROIs adjacent to adhesions was subtracted from the raw fluorescence intensities prior to analysis. Time elapsed between the onset of GFP-paxillin intensity increase and the appearance of associated mCh-α-actinin was quantified for each adhesion.

### Fluorescence recovery after photobleaching (FRAP)

Control and dynamin2-depleted U2-OS cells expressing low levels of GFP-actin under the control of a truncated CMV promoter [28] were plated on 30 μg/ml laminin for 4 hours. FRAP experiments were performed using an LSM780 inverted microscope (Zeiss, Jena, Germany) equipped with a 63x, 1.4 n.a. Plan-APOCHROMAT objective and GaSaP detector. Selected areas (1.5 μm×7 μm) of protruding lamellipodia were bleached (30 iterations at full laser power at 488 nm, 30 mW argon laser) and then imaged for 75 s at 1 frame/s. Images were corrected for photobleaching and background, and the intensities of GFP-actin in bleached regions measured over time using ImageJ software (NIH, Bethesda, MD). Intensities were normalized to the mean GFP-actin fluorescence intensity of the last five frames before bleaching. Data were fit to one-phase association curve using Prism 5.0 (GraphPad Software, La Jolla, CA).

### Statistical analysis

Data were analyzed using a two-tailed Student's *t* test, unequal variance (Prism 5.0); significance values specific for each analysis are indicated in the figure legends. All experiments were performed a minimum of three times, except some rescue experiments and analyses of focal adhesions that were performed in duplicate, as indicated in the figure legends.

## Results

### Dynamin2 regulates lamellar actomyosin and retrograde flow

Previous work implicated dynamin2 in regulating actomyosin networks in osteosarcoma cells, podocytes and epithelial cells [18], [20], [21]. To determine how dynamin2, which is enriched in lamellipodial actin networks [19], [29]–[31], influences lamellar actomyosin, we observed the formation of nascent actomyosin in living U2-OS cells lacking up to ∼90% of endogenous dynamin2. U2-OS cells assemble several actomyosin structures, including transverse arcs aligned parallel to the cell edge [2], [3], [32], [33]. Transverse arcs are proposed to arise, in part, from lamellipodial F-actin and the early steps in their assembly occur near the boundary between the lamellipod and the lamellum [2], [4]–[6]. Transverse arcs flow retrogradely and interact with focal adhesions and dorsal fibers oriented orthogonal to the arcs [2], [33]–[35].

**Figure 1:**
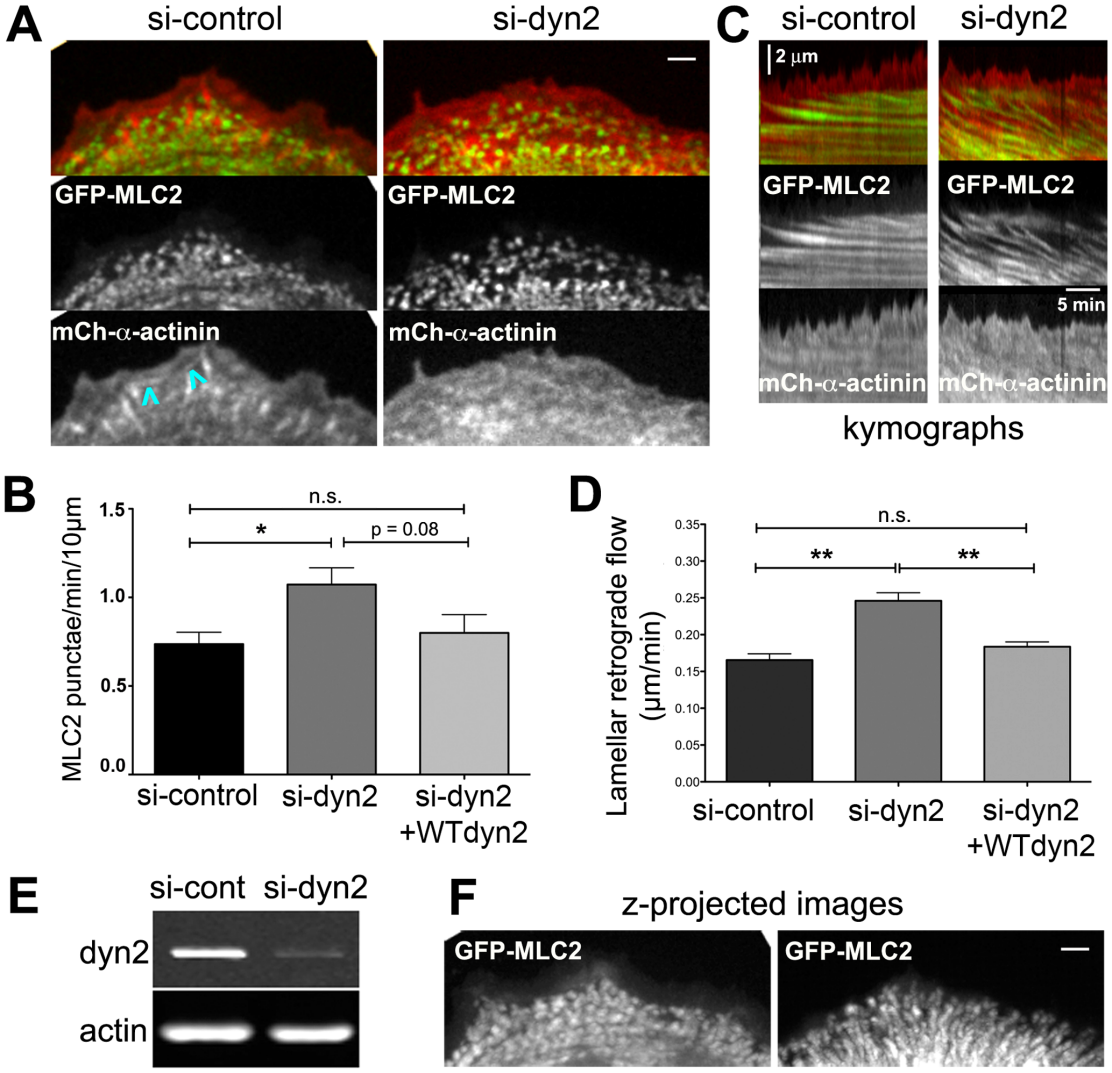
Dynamin2 influences actomyosin assembly in U2-OS cells. (A) Images from movies of control and dynamin2-depleted U2-OS cells transiently expressing GFP-MLC2 and mCh-α-actinin as indicated (see Movie S1). Bar is 2 μm. Blue arrowheads identify curved, α-actinin-decorated cables that transiently engage focal adhesions. (B) Bar graph indicating the mean number of nascent GFP-MLC2 punctae formed along 10 μm of the cell edge/min in cells treated with control siRNA or dyn2 siRNA, with or without expression of mCh-WT dyn2. *n* = 15 (si-control), *n* = 13 (si-dyn2) and *n* = 7 (si-dyn2+dyn2-WT) regions (5–14 cells/condition); * p<0.05, n.s., not significant. Data for si-control and si-dyn2 are from three individual experiments; data for si-dyn2+dyn2-WT are from two independent experiments. (C) Representative kymographs generated from pixel-wide segments orthogonal to the cell edge of movies of control and dyn2-depleted cells expressing GFP-MLC2 and mCh-α-actinin as indicated. (D) Bar graph indicating mean lamellar retrograde flow in cells treated with control siRNA or dyn2 siRNA, with and without expression of mCh-WT-dyn2. *n* = 56 (si-control), *n* = 46 (si-dyn2) and *n* = 38 (si-dyn2+dyn2-WT) kymographs (4-5 cells/condition); ** p<0.005, n.s., not significant. Data for si-control and si-dyn2 are representative of at least five individual experiments; data for WT-dyn2 rescue is representative of duplicate independent experiments. (E) Western blot showing reduced dynamin2 protein in whole cell lysates of si-dyn2-treated cells compared with control siRNA-treatment; lanes were loaded with equivalent numbers of cells and actin is the loading control. (F) Brightest point projected images generated from 40 frames of the GFP-MLC2 movie from panel A (Movies S1) highlight differences in actomyosin retrograde flow patterns in control and dyn2-depleted cells. Error bars indicate the s.e.m.

To observe assembly of actomyosin arcs, we acquired time-lapse movies of cells expressing GFP-myosin light chain 2 (GFP-MLC2), a marker for non-muscle myosin II, and mCherry (mCh)-α-actinin, a marker for actin filaments (Fig. 1A, Movie S1). Nascent non-muscle myosin II first appeared near the lamellipod-lamellum boundary as small uniformly sized punctae of GFP-MLC2, approximately 0.6 μm in width. GFP-MLC2 punctae appeared at a rate of ∼0.8 punctae/min/10 μm of cell edge (Fig. 1B) and flowed retrogradely (Fig. 1C). GFP-MLC2 punctae acquired a uniform size and regular spacing as they coalesced as arcs aligned parallel to the cell edge. On the other hand, the spatiotemporal distribution of mCh-α-actinin in lamellipodial and lamellar actin networks was complex. mCh-α-actinin was enriched in lamellipodia where it flowed rearward at a fast rate (discussed below, Fig. 3). mCh-α-actinin accumulated at focal adhesions and transiently decorated cable-like structures that appeared near the boundary between the lamellipod and lamellum; the cables often bridged adjacent focal adhesions as they flowed slowly rearward (Fig. 1A, arrowheads and Movie S1) [7], [36]. As lamellar actomyosin matured, the distribution of mCh-α-actinin along arcs became discontinuous and interspersed with punctae of GFP-MLC2, as described previously [2].

Our previous work using fixed cells demonstrated that aberrant actomyosin arcs formed in U2-OS cells treated with two different siRNAs targeting dynamin2 [21]. We show here that depleting 89%±2.6 s.e.m. of endogenous dynamin2 (Fig. 1E) perturbed nascent arcs as they formed in U2-OS cells (Movie S1). GFP-MLC2 punctae appeared more frequently in dyn2-depleted cells (Fig. 1B) and some punctae acquired irregular shapes, either expanding in size or merging with adjacent punctae as they flowed rearward (Movie S1). Lamellar actomyosin flowed retrogradely ∼1.5-fold faster in dyn2-depleted cells compared to that in control siRNA-treated cells (Fig. 1D). Importantly, the rate of appearance of GFP-MLC2 punctae and the rate of lamellar actomyosin retrograde flow was restored in dyn2-depleted cells expressing wild type (WT) rat mCh-dynamin2 (Fig. 1B,D). Finally, in contrast with the uniformly radial pattern of retrograde flow observed in control cells, retrograde flow in some dynamin2-depleted cells appeared irregular (Movie S1). The aberrant dynamics of GFP-MLC2 punctae in dynamin2-depleted cells was apparent by kymography (Fig. 1C) and in brightest point z-projected images generated from 40 frames of the time-lapse movies (Fig. 1F).

The distribution of mCh-α-actinin in dynamin2-depleted cells was generally similar to that observed in control siRNA-treated cells. As in control cells, mCh-α-actinin in dynamin2-depleted cells was distributed within lamellipodia and decorated lamellar actin structures that, like GFP-MLC2 punctae, flowed rearward at a rapid rate (Fig. 1A; Movie S1). Interesting, mCh-α-actinin was not enriched at focal adhesions formed near the cell periphery, even though focal adhesions formed near the leading edge of dynamin2-depleted cells (see below), suggesting that dynamin2 might influence protein composition at newly formed focal adhesions. Taken together, these data indicate that dynamin2 influences the assembly and retrograde flow of lamellar actomyosin arcs in U2-OS cells. Because dynamin2 is enriched in lamellipodial F-actin networks [19], [29]–[31] (and see below), we hypothesize that dynamin2 influences assembly and dynamics of actomyosin arcs via its actions on lamellipodial actin filaments.

To determine whether or not the effects of dynamin2 depletion on actomyosin arcs might result from alterations in endocytic traffic, we quantified internalization of two plasma membrane receptors in control- and dyn2-siRNA-treated U2-OS cells. The rate of internalization of transferrin receptor and β1-integrin were similar in control and dynamin2-depleted cells (Fig. S1A). The cell surface pool of transferrin receptor was not significantly altered in dynamin2-depleted cells, but surface levels of β1-integrin were slightly decreased (Fig. S1B). Although the lack of a significant effect on receptor internalization by dynamin2-depleted cells was unexpected, redundant endocytic pathways likely exist [22], [37]. Endocytosis also could be executed in dyn2-depleted U2-OS cells by dynamin1 (Fig. S1C), by residual dynamin2 or by an unidentified compensatory pathway. We conclude that the effects on actin networks observed upon depleting dynamin2 in U2-OS cells do not result secondarily from perturbations in endocytosis. Other experiments in which mutant dynamin2 proteins that are competent for endocytosis, but do not fully restore actomyosin arcs when expressed in dynamin2-depleted cells (see below), lend additional support to this conclusion.

### Dynamin2 is enriched in the distal region of advancing lamellipodia

**Figure 2:**
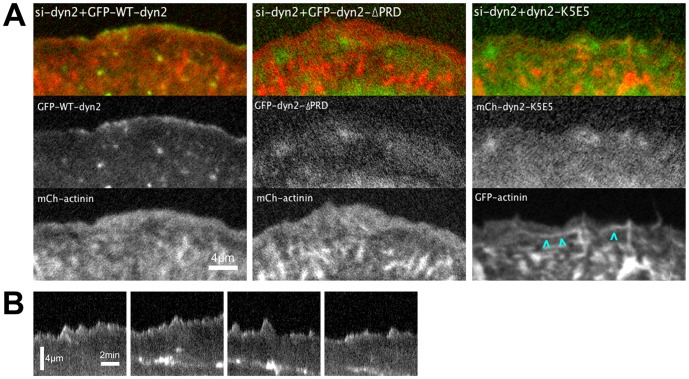
Dynamin2 is targeted to the distal lamellipod via interactions of the dynamin2 stalk and the dynamin2 PRD. (A) Images from movies of dyn2-depleted U2-OS cells transiently expressing either GFP-WT-dyn2 or GFP-dyn2-ΔPRD, as indicated, and mCh-α-actinin, or mCh-dyn2-K_5_E_5_ and GFP-α-actinin, as indicated (see Movie S2); cyan arrowheads highlight GFP-α-actinin-decorated cables induced in cells expressing mCh-dyn2-K_5_E_5_. The images of mCh-dyn2-K_5_E_5_ and GFP-α-actinin were pseudocolored αs green and red, respectively, to correspond with the other images displayed in this panel. (B) Representative kymographs obtained from pixel-wide lines orthogonal to the leading edge of dynamin2-depleted cells expressing GFP-WT-dyn2 depicting the spatial and temporal distribution of GFP-WT-dyn2. Scale bars: as indicated.

To test the hypothesis that dynamin2 influences lamellar actomyosin via effects on lamellipodial F-actin networks, we localized rat GFP-WT-dynamin2aa (GFP-WT-dyn2) in live U2-OS cells depleted (or not) of endogenous dynamin2. As expected, GFP-WT-dyn2 associated with lamellipodial actin networks and with dynamic foci within lamella when expressed at low amounts in dyn2-depleted cells (Fig. 2A, left panels, Movie S2) [19], [30], [31]. Kymographs taken from the time-lapse movies revealed that GFP-WT-dyn2 was selectively enriched in the distal-most region of advancing protrusions; retracting regions of the lamellipod were nearly devoid of GFP-WT-dyn2 (Fig. 2B, Movie S2). Within lamellar regions, GFP-WT-dyn2 also appeared at transient, diffraction-limited punctae that are likely sites of clathrin-mediated endocytosis (Movie S2) [38], [39]. In contrast with GFP-WT-dyn2, mCh-dynamin1 associated with diffraction-limited punctae within the cytoplasm, but was not associated with lamellipodial actin networks (Fig. S2). The dynamic association of GFP-WT-dyn2 with the distal-most region of advancing lamellipodial F-actin suggests that dynamin2 acts on a subset of actin filaments that comprise such networks.

Interactions of its C-terminal, proline-rich domain (PRD) have been implicated in targeting dynamin2 to lamellipodia [19], [29], [31], a result we confirmed in live cells using a mutant dynamin2 lacking the PRD (Fig. 2A, center panels; Movie S3). Dynamin2 also directly binds actin filaments *in vitro* via a basic region in the dynamin2 stalk [18]. To test whether dynamin2 localized to lamellipodia actin networks via direct interactions with F-actin, we introduced mutations in the dynamin2 stalk (five K to E mutations at amino acids 414, 415, 419, 421 and 426; (mCh-dyn2-K_5_E_5_)), creating a mutant dynamin2 protein reported to have reduced affinity for actin filaments [18]. When expressed at low amounts in U2-OS cells previously depleted of endogenous dynamin2, mCh-dyn2-K_5_E_5_ distributed diffusely throughout the lamellipod and lamellum and was not enriched at the leading edge of advancing protrusions (Fig. 2A, right panels; Movie S4). Thus, recruitment of dynamin2 to the distal lamellipod depends on basic amino acid residues in the dynamin2 stalk domain that interact with actin filaments. Expression of mCh-dyn2-K_5_E_5_ also induced formation of GFP-α-actinin-decorated cable-like structures that formed from retracting lamellipodial protrusions and flowed rearward into the lamellum (Fig. 2A, arrowheads; Movie S4). This result suggests that F-actin binding by dynamin2 regulates actin filament bundling. As was observed with GFP-dyn2-ΔPRD, occasional regions behind the leading edge became transiently enriched in mCh-dyn2-K_5_E_5_ (Movie S4). Such regions of increased mCh-dyn2-K_5_E_5_ fluorescence likely result from locally increased cytoplasmic volume. Taken together, we conclude that interactions by both the C-terminal PRD and basic regions within its stalk are required to target dynamin2 to the distal edge of advancing lamellipodial protrusions. The enrichment of WT-GFP-dynamin2 with lamellipodial F-actin, but not with lamellar actin networks, supports the hypothesis that dynamin2 acts on lamellipodial F-actin to influence formation of lamellar actomyosin.

Dyn2-K5E5 is reported to support endocytosis [18]; however, mCh-dyn2-K_5_E_5_ was not apparent at dynamic punctate structures characteristic of endocytic pits as observed in movies of cells expressing GFP-WTdyn2 (compare Movie S2 and Movie S4). To determine if dyn2-K_5_E_5_ accumulates at endocytic sites, we examined fixed cells expressing GFP-WTdyn2, GFP-dyn2-ΔPRD or GFP-dyn2-K_5_E_5_ and immunolabeled with antibodies to AP2, the clathrin adaptor complex that associates with membrane receptors at the plasma membrane (Fig. S3). As observed in live cells, GFP-dyn2-K_5_E_5_ was diffusely cytoplasmic and detected in lamellipodia of fixed cells (Fig. S3A). Unlike GFP-WTdyn2, which was enriched at most AP2-positive punctae, GFP-dyn2-K_5_E_5_ was only faintly apparent at some punctate structures labeled with anti-AP2 (Fig. S3A, B). GFP-dyn2-ΔPRD, which is not targeted to sites of endocytosis, was not detected at AP2-positive punctae. Thus, GFP-dyn2-K_5_E_5_ was weakly targeted to endocytic sites compared to GFP-WT-dyn2. To further explore the participation of GFP-dyn2-K_5_E_5_ at endocytic sites, we examined the dynamics of GFP-WT-dyn2 and GFP-dyn2-K_5_E_5_ at the ventral plasma membrane of live cells using total internal reflection fluorescence microscopy. Both GFP-WTdyn2 and GFP-dyn2-K_5_E_5_ were detected at punctate structures at the ventral cell surface, however, GFP-dyn2-K_5_E_5_ punctae were considerably less dynamic than those identified by GFP-WT-dyn2 (Movie S5 and Fig. S3C). Although the nature of the endocytic structure associated with dynamin2-containing punctae at the plasma membrane was not determined, the distinct dynamic behaviors exhibited by GFP-dyn2-K_5_E_5_ and GFP-WTdyn2 strongly suggest that their endocytic activities may differ. It is possible that F-actin binding by dynamin2 regulates its dynamics at sites of endocytosis rather than membrane fission activity. Nonetheless, additional experiments are required to determine how dynamin2-K_5_E_5_ participates in endocytosis.

### Dynamin2 influences lamellipodial actin network organization and dynamics

**Figure 3:**
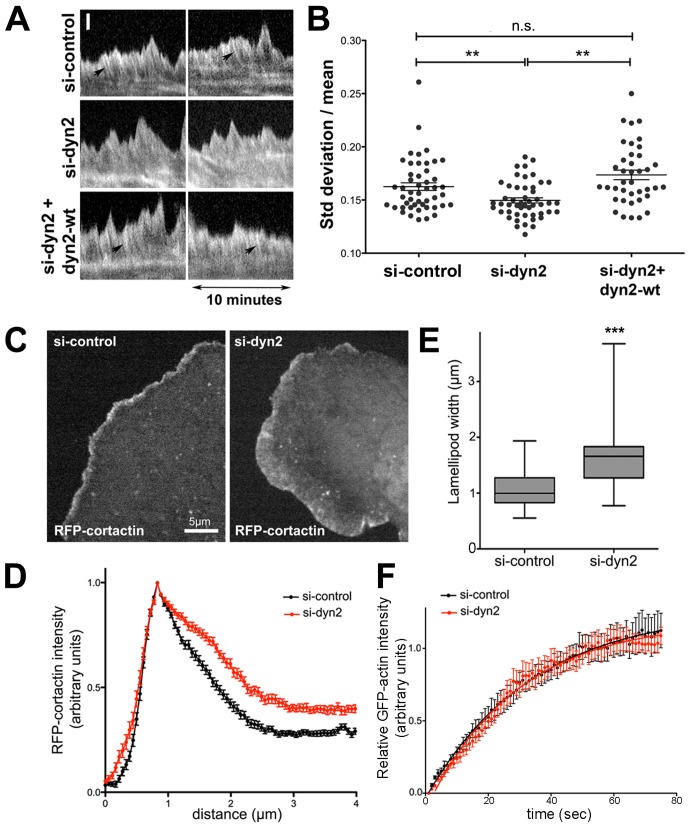
Dynamin2 influences the organization of lamellipodial actin filaments and restricts lamellipodium width. (A) Representative kymographs obtained from timelapse movies of GFP-α-actinin in lamellipodia of U2-OS cells treated with control siRNAs (si-control) or dynamin2 siRNAs (si-dyn2) with and without expression of mCh-WT-dyn2 (si-dyn2+dyn2-WT). Arrows denote stripes in the kymographs that represent regions of lamellipodial F-actin enriched in GFP-α-actinin (see Movie S6). Scale bar: 1 μm. (B) Graph depicting the variation in GFP-α-actinin fluorescence intensity plotted as SD/mean within the lamellipodial region of kymographs from si-control-treated, si-dyn2-treated, and si-dyn2-treated, mCh-WT-dyn2 expressing U2-OS cells. *n* = 48 (si-control), *n* = 49 (si-dyn2) and n = 42 (si-dyn2+dyn2-WT) regions (5-6 cells/condition); ** p<0.005; ns, not significant. Data for si-control and si-dyn2 are representative of results from more than five independent experiments; data from si-dyn2+dyn2-WT are representative of results from duplicate experiments. (C) Images from movies of si-control-treated and si-dyn2-treated U2-OS cells expressing RFP-cortactin. (D) RFP-cortactin fluorescence intensity distribution along line scans placed orthogonal to the cell edge in protruding regions of lamellipodia (mean of 52 (si-control) and 47 (si-dyn2) line scans/condition). Data are representative of duplicate experiments. (E) Box and whisker plot of lamellipodium width as measured from the half maximal RFP-cortactin intensity along line scans (52 (si-control) and 47 (si-dyn2) line scans/condition; data are representative of duplicate experiments. ***, p<0.001. (F) Graphs depicting the normalized mean + s.e.m. for recovery of GFP-actin fluorescence after photobleaching in lamellipodia of si-control-treated and si-dyn2-treated U2-OS cells. Data are fit to a one-phase exponential association curve. *n* = 10 (si-control) and *n* = 12 (si-dyn2); data are representative of three independent experiments.

To determine how dynamin2 influences lamellipodial actin networks, we examined the spatiotemporal dynamics of GFP-α-actinin within lamellipodia using images collected at higher spatial and temporal resolution. GFP-α-actinin offers unique advantages as a probe of actin networks because α-actinin associates with a subset of actin filaments within lamellipodial networks implicated in formation of lamellar actomyosin [2], [33] and actin filament crosslinking by α-actinin directs formation and maturation of nascent focal adhesions [10]. In control siRNA-treated cells, GFP-α-actinin was enriched in the distal lamellipod and flowed rearward at a high rate expected for a component of lamellipodial F-actin (Movie S6). Interestingly, the spatiotemporal distribution of GFP-α-actinin within lamellipodial actin networks of control cells was non-homogeneous. When visualized using kymographs of the lamellipod, GFP-α-actinin appeared as distinct, diagonally oriented stripes (Fig. 3A, arrows). We interpret these stripes as subsets of α-actinin-enriched actin filaments undergoing retrograde flow. The intensity of GFP-α-actinin fluorescence was most intense at the cell periphery, becoming less intense closer to the boundary of the lamellipod and lamellum. The decreased GFP-α-actinin intensity toward the rear of the lamellipod of control cells contributed to the appearance of a distinct boundary between the lamellum and proximal lamellipod where actin network retrograde flow slowed abruptly.

In contrast, the spatiotemporal distribution of GFP-α-actinin within lamellipodia of dynamin2-depleted cells appeared homogeneous compared to that in control cells; distinct stripes of GFP-α-actinin were less prominent and the boundary between the lamellipod and lamellum was diffuse (Fig. 3A and Movie S6). When viewed in timelapse movies, GFP-α-actinin-decorated F-actin appeared to flow continuously from the lamellipod to the lamellum. To quantitatively assess the spatiotemporal distribution of GFP-α-actinin in lamellipodia, we measured the variation in GFP-α-actinin fluorescence intensity over time within the lamellipodial region (Fig. 3B). The temporal variation in GFP-α-actinin fluorescence (determined as the standard deviation in GFP-α-actinin fluorescence/mean fluorescence intensity; see Experimental Methods) within lamellipodia was significantly decreased in dynamin2-depleted cells compared to that in control siRNA-treated cells. Importantly, expression of mCh-WT-rat dynamin2 in dyn2-depleted cells increased the variation of GFP-α-actinin fluorescence to that of control cells, thus restoring the spatiotemporal distribution of lamellipodial GFP-α-actinin (Fig. 3B). We conclude that the different spatiotemporal distributions of GFP-α-actinin within lamellipodia of control and dyn2-depleted cells reflect distinct actin filament organizations within lamellipodia under these two conditions. Moreover, dynamin2 at the distal lamellipod acts to spatially segregate the lamellipodium and lamellum.

As a second test of dynamin2 effects on lamellipodial actin networks, we localized cortactin, a component of lamellipodial actin networks, in control and dynamin2-depleted cells. Immunolabeling for endogenous cortactin along the periphery of fixed U2-OS cells was discontinuous, making it difficult to quantify cortactin's distribution (Fig. S4A), therefore, we examined RFP-cortactin in living U2-OS cells (Fig. 3C). Line scans of RFP-cortactin intensity orthogonal to the cell edge of protruding regions of control U2-OS cells revealed a narrow band ∼1 μm at half-width of cortactin concentrated at the leading edge (Fig. 3D, E). In contrast, the distribution of RFP-cortactin within protruding lamellipodia in dynamin2-depleted cells was significantly broader (Fig. 3D, E). Thus, consistent with a role for dynamin2 in organizing lamellipodial actin filaments, dynamin2 acts to restrict lamellipodial width.

Finally, to determine if dynamin2 influences dynamics of lamellipodial F-actin, we compared rates of actin filament turnover using fluorescence recovery after photobleaching (FRAP) analysis of GFP-actin. Recovery of GFP-actin of the distal lamellipod was identical in control and dynamin2-depleted U2-OS cells (Fig. 3F), indicating that dynamin2 does not influence actin filament dynamics within lamellipodia. Taken together, the results of these studies support the hypothesis that dynamin2 acts on filaments of the distal lamellipod to influence the formation and dynamics of lamellar F-actin networks.

### Dynamin2 influences the linkage of flowing lamellar F-actin at adhesions

**Figure 4:**
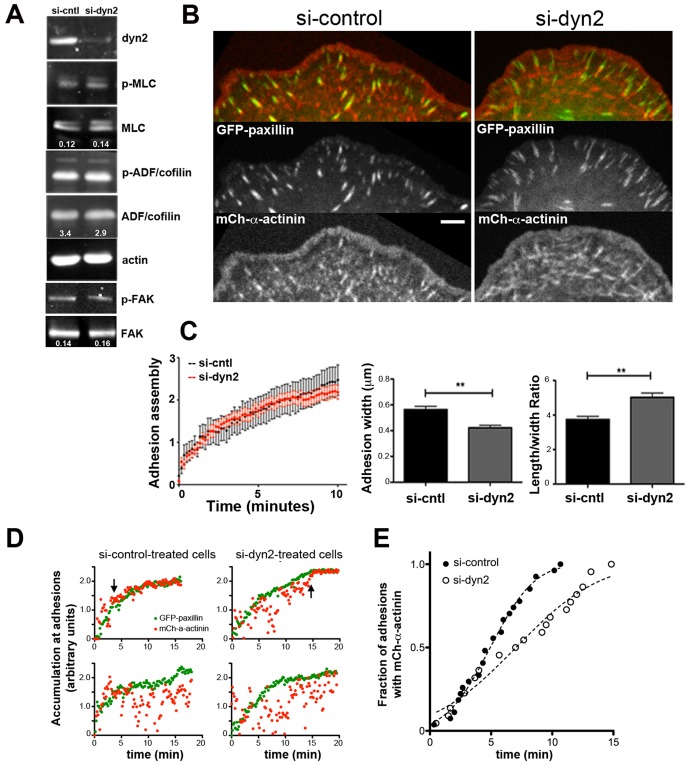
Dynamin2 promotes coupling of F-actin and adhesions. (A) Western blots depict levels of total and phosphorylated MLC2, ADF/cofilin and FAK in si-control and si-dyn2-treated U2-OS cells. Images were obtained using a Li-COR quantitative imaging system and numbers denote the ratio of phospho-to-total protein for each pair; actin is the loading control. (B) Representative images from movies of si-control-treated and si-dyn2-treated U2-OS cells expressing GFP-paxillin and mCh-α-actinin (see Movie S7). Bar = 5 μm. (C) Rate of assembly of GFP-paxillin at focal adhesions plotted as mean + s.e.m. ln (I_t_/I_0_) vs. time as described in experimental methods. *n* = 19 (si-control) and *n* = 46 (si-dyn2) adhesions. Bar graphs depict the width and length/width ratio of newly formed adhesions at the leading edge of control and dyn2-depleted cells. *n* = 53 (si-control) and *n* = 48 (si-dyn2) adhesions, ** p<0.005. (D) Graphs depict the accumulation of GFP-paxillin (green) or mCh-α-actinin (red) at individual adhesions in si-control-treated cells (left panels) and si-dyn2-treated cells (right panels). Plotted is ln(I_t_/I_0_) vs time; data for mCh-α-actinin was normalized to align with the GFP-paxillin data. Upper panels depict examples of adhesions in which mCh-α-actinin accumulated with GFP-paxillin during the time of observation period as indicated by black arrows; lower panels depict examples of adhesions in which mCh-α-actinin did not accumulate with GFP-paxillin during the observation period. (E) Graph depicts the cumulative distribution of mCh-α-actinin-containing adhesions over time. Plotted is the fraction of adhesions with mCh-α-actinin over time; data does not include adhesions for which mCh-α-actinin did not accumulate during the time of observation (see text). Dotted lines indicate the fit of each dataset to a Gaussian distribution; different curves are appropriate for each data set, p<0.0001. *n* = 20 (si-control) and *n* = 18 (si-dyn2) adhesions; data are representative of two independent experiments.

The increased rate of lamellar retrograde flow observed in dynamin2-depleted cells could arise via several mechanisms. While FRAP of GFP-actin did not support a role for dynamin2 in regulating lamellipodial F-actin dynamics, dynamin2 could influence signaling pathways that regulate contractility or adhesions to control lamellar retrograde flow. Alternatively, dynamin2 could influence coupling of F-actin at adhesions. To determine if dynamin2 influences signaling that regulates contractility or actin dynamics, we quantified the levels of phosphorylated MLC2 and ADF/cofilin, two factors generated downstream of active RhoA, in control and dynamin2-depleted cells (Fig 4A). Neither the total amounts of MLC2 or ADF/cofilin, nor the amounts of phosphorylated form of each protein, were altered in lysates of dyn2-depleted U2-OS cells compared with lysates of control, siRNA-treated U2-OS cells (Fig. 4A). Cellular levels of focal adhesion kinase (FAK) and phospho-FAK or of VAV2, a Rac effector and Rac guanine nucleotide exchange factor, respectively, that interact with dynamin2 [31], [40] were also unchanged in dyn2-depleted U2-OS cells (Fig. 4A and Fig. S4B). Although the possibility that RhoA and Rac are activated locally and transiently via a dyn2-dependent process is not ruled out, dynamin2 does not influence wholesale signaling to key actin cytoskeletal regulators.

Engagement of F-actin at focal adhesions is thought to slow F-actin retrograde flow, limit the width of the lamellipodium and spatially segregate the lamellipod and lamellum [6], [7], [9], [41]–[43]. To determine if dynamin2 influences engagement of flowing F-actin at focal adhesions, we first examined assembly and morphology of focal adhesions using GFP-paxillin (Fig. 4B; Movie S7). Whereas the rates of new adhesion assembly were identical in control and dyn2-depleted cells (Fig. 4C, left panel), the morphology of newly formed adhesions differed (Fig. 4C, right panels). New adhesions in control cells elongated rapidly and became wider as they matured. In contrast, nascent focal adhesions in dyn2-depleted cells elongated rapidly, but remained thin (Fig. 4C), exhibiting a higher length/width ratio compared to adhesions in control cells. Thus, dynamin2 influenced adhesion morphology, but not the rate of adhesion assembly.

α-Actinin is one molecular link between adhesions and flowing actin filaments. α-Actinin organizes lamellipodial actin filaments as nascent adhesions form and also accumulates on maturing adhesions [10], [44]–[46]. To determine whether dynamin2 influences the linkage between adhesions and flowing F-actin, we quantified the relative appearance of mCh-α-actinin and GFP-paxillin as adhesions formed in control and dynamin2 depleted cells (Fig. 4D; Movie S7). Most new adhesions in control cells acquired mCh-α-actinin at their proximal end within 10 min of the appearance of GFP-paxillin (Fig. 4D, upper left panel, and Fig. 4E); 13% of newly formed adhesions in control cells did not acquire mCh-α-actinin within the duration of the movie (Fig. 4D, lower left panel) and, thus, were not included in the cumulative analysis shown in Fig. 4E. In contrast, mCh-α-actinin accumulated at adhesions in dynamin2-depleted cells more slowly than in control cells (Fig. 4D, upper right panel and Fig. 4E) and mCh-α-actinin did not associate with 41% of newly formed adhesions within the duration of the timelapse imaging data (Fig. 4D, lower right panel). Consistent with these results, mCh-α-actinin-decorated structures streamed rearward past adhesions in dynamin2-depleted cells (Movie S7). Thus, the delayed recruitment of mCh-α-actinin at adhesions in dynamin2-depleted cells supports the idea that dynamin2 regulates engagement of flowing lamellar F-actin at adhesions. We hypothesize that dynamin2 of the distal lamellipod remodels lamellipodial actin filaments to promote interactions at adhesions.

### Actin filament remodeling by dynamin2 requires interactions with actin filaments, interactions of the PRD and finely tuned GTP hydrolysis

**Figure 5:**
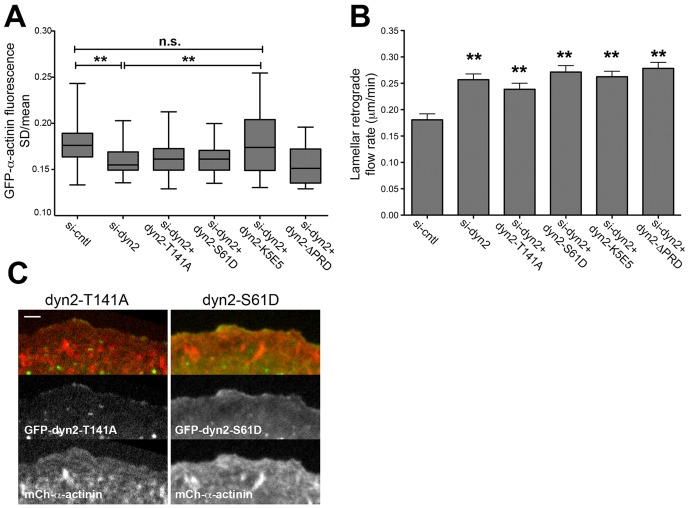
Regulation of lamellipodial and lamellar actin networks depends on F-actin binding by the dynamin2 stalk, the dynamin2 PRD and finely tuned GTP hydrolysis. (A) Box and whisker plots of the standard deviation/mean intensity (SD/mean) of lamellipodial GFP-α-actinin fluorescence from kymographs of the lamellipodial region of si-control-treated and si-dyn2-treated U2-OS cells, with and without expression of mCh-dyn2-T141A, mCh-dyn2-S61D, mCh-dyn2-K_5_E_5_, or mCh-dyn2-ΔPRD, as indicated. *n* = 88 (si-control), *n* = 57 (si-dyn2), *n* = 73 (si-dyn2+dyn2-T141A), *n* = 50 (si-dyn2+dyn2-S61D), *n* = 55 (si-dyn2+dyn2-K_5_E_5_) and *n* = 22 (si-dyn2+dyn2-ΔPRD) regions from 3-9 cells/condition). ** p<0.005; ns, not significant. Data are representative of that from two independent experiments. (B) Bar graphs depict mean rate of lamellar retrograde flow determined from analysis of the flow of GFP-MLC2-containing lamellar actomyosin structures in kymographs of movies from si-control-treated and si-dyn2-treated U2-OS cells, with and without expression of mCh-dyn2-T141A, mCh-dyn2-S61D, mCh-dyn2-K_5_E_5_, or mCh-dyn2-ΔPRD, as indicated. Error bars indicate the s.e.m. *n* = 25 (si-control), *n* = 33 (si-dyn2), *n* = 52 (si-dyn2+dyn2-T141A), *n* = 33 (si-dyn2+dyn2-S61D), *n* = 58 (si-dyn2+dyn2-K_5_E_5_) and *n* = 24 (si-dyn2+dyn2-ΔPRD) regions from 4-5 cells/condition). ** p<0.005. (C) Images are a frame from timelapse movies of si-dyn2-treated cells expressing GFP-dyn2-T141A (left panels) or GFP-dyn2-S61D (right panels), together with mCh-α-actinin, as indicated (see Movie S9). Bar = 2 μm.

To determine the biochemical activities required by dynamin2 to influence actin networks, we expressed mutant forms of dynamin2 in dyn2-depleted cells and assessed their ability to restore lamellar retrograde flow and the spatiotemporal dynamics of GFP-α-actinin in lamellipodia. Because the C-terminal PRD targets dynamin2 to the distal lamellipod, it was not surprising that mCh-dyn2-ΔPRD neither restored the spatiotemporal distribution of lamellipodial GFP-α-actinin nor the rate of lamellar retrograde flow in dyn2-depleted cells (Fig. 5A,B). In addition, nascent GFP-MLC2 punctae in cells expressing mCh-dyn2-ΔPRD appeared uncontrolled and misshaped, similar to those in dynamin2-depleted cells (Movie S8). These findings highlight the role of PRD interactions for dynamin2-dependent activities on actin filaments.

To determine if interactions of dynamin2 and actin filaments was required to influence actin networks, we expressed the actin-binding deficient mutant protein, mCh-dyn2-K_5_E_5_, in dyn2-depleted cells. Although mCh-dyn2-K_5_E_5_ was diffusely distributed within lamellipodia (Fig. 2C), the variation of GFP-α-actinin in lamellipodia, as assessed from the variation of GFP-α-actinin intensity (SD/mean), was restored to that observed in control cells when mCh-dyn2-K_5_E_5_ was expressed in dynamin2-depleted cells (Fig. 5A). However, as discussed above, the prominent α-actinin-decorated cables induced in cells expressing mCh-dyn2-K_5_E_5_ (Fig. 2; Movie S4) likely contributed to the increased variation in GFP-α-actinin fluorescence. On the other hand, the rate of lamellar retrograde flow remained elevated in dynamin2-depleted cells expressing mCh-dyn2-K_5_E_5_ (Fig. 5B). Additionally, as was observed in dynamin2-depleted cells, GFP-MLC2 punctae appeared uncontrolled and acquired aberrant shapes (Movie S8). Thus, mCh-dyn2-K_5_E_5_ did not fully restore actin network organization or dynamics to dyn2-depleted cells, underscoring a role for direct interactions of dynamin2 and actin filaments within the distal lamellipod.

GTP hydrolysis by dynamin2 and cortactin remodeled bundled actin filaments *in vitro* [21]; moreover, dynamin2 GTPase activity was implicated in controlling F-actin dynamics at sites of clathrin-mediated endocytosis [47] and in regulating apical actomyosin in epithelial cells [20]. Therefore, we asked if the rate of GTP hydrolysis by dynamin2 influenced lamellipodial and lamellar actin networks. Two mutant dynamin2 proteins, dyn2-T141A and dyn2-S61D, which exhibit increased and decreased, respectively, rates of basal and lipid-stimulated GTP hydrolysis [48] were expressed in dynamin2-depleted U2-OS cells. Like GFP-WT-dyn2, GFP-dyn2-T141A and GFP-dyn2-S61D localized at the distal edge of advancing lamellipodial protrusions (Fig. 5C and Movie S9). Nonetheless, neither GTP-hydrolysis defective mutant dynamin2 protein restored lamellipodial filament organization, as assessed from the spatiotemporal distribution of GFP-α-actinin (SD/mean) (Fig. 5A), or lamellar retrograde flow (Fig. 5B) to dynamin2-depleted cells. Finally, as was observed in dynamin2-depleted cells, the pattern of retrograde flow was irregular, particularly in cells expressing GFP-dyn2-T141A (Movie S8). Thus, we conclude that the rate of GTP hydrolysis by dynamin2 associated with lamellipodial actin networks influences lamellipodial actin filament organization and lamellar retrograde flow. Because dyn2-T141A supports receptor mediated endocytosis [48], but did not fully restore dynamin2-dependent activities on actin filaments, reinforces the conclusion that dynamin2-dependent remodeling of lamellipodial actin networks is independent of dynamin2 function in receptor-mediated endocytosis.

## Discussion

We report that dynamin2 of the distal lamellipod of U2-OS cells acts on lamellipodial actin filaments and influences the assembly and dynamics of lamellar actomyosin situated several microns away. Considerable evidence has implicated dynamin2 in regulating the actin cytoskeleton, but the physiologic significance of its action remains unknown [11], [12], [20], [21], [29], [49], [50]. Our results implicate dynamin2 in remodeling filaments within dendritic actin networks that give rise to lamellar actomyosin and engage F-actin flow at cell matrix adhesions. In this way, dynamin2 generates a coherent actin cytoskeletal network. Actin filament remodeling by dynamin2 depended on its rate of GTP hydrolysis, interactions via its PRD and F-actin binding by the dynamin2 stalk region.

Dynamin2 associated selectively with actin filaments situated at the distal-most edge of advancing protrusions. As expected from previous findings [19], the dyn2 PRD targeted dynamin2 to the lamellipod. Interestingly, direct interactions with F-actin via the dyn2 stalk are also involved in targeting dynamin2 to the distal lamellipod. The dynamin2 stalk might preferentially bind distal actin filaments via interactions with ATP-actin subunits that are enriched in newly assembled F-actin (Pollard and Borisy, 2003). Alternatively, as proposed for selective recruitment of Arp2/3 complex to actin filaments adjacent to the plasma membrane [51], interactions of the dynamin2 stalk with F-actin could depend on structural features that arise from mechanical factors acting on filaments elongating close to the membrane. Cortactin, which binds the dynamin2 PRD, actin filaments and Arp2/3 complex, may refine interactions of dynamin2 with actin filaments subjacent to advancing membranes, perhaps by anchoring dynamin2 at or near branched filament junctions.

Although we did not directly interrogate actin filament organization in U2-OS cells, several findings support the idea that dynamin2 influences actin filament architecture within lamellipodia. First, α-actinin-decorated F-actin was selectively enriched in regions of the distal lamellipod of control cells, but was homogeneously distributed in lamellipodia of cells lacking dynamin2. The non-homogeneous spatiotemporal distribution of mCh-α-actinin in lamellipodia of U2-OS cells is consistent with existence of a distinct subset of α-actinin-decorated actin filaments specified by dynamin2. Second, prominent bundles formed as lamellipodial protrusions retracted in dynamin2-depleted cells expressing a mutant dynamin2 defective in F-actin binding (mCh-dyn2-K_5_E_5_), indicating a role for direct F-actin binding by dynamin2 for filament remodeling within lamellipodia. Interactions of dynamin2 and cortactin likely promote filament bundling in cells expressing mCh-dyn2-K_5_E_5_ [21]. Finally, the dimensions of lamellipodial actin networks, as defined by the distribution of cortactin, depended on dynamin2. Taken together, we conclude that through its actions on lamellipodial F-actin, dynamin2 remodels select actin filaments to specify crosslinking by α-actinin. Although α-actinin can crosslink actin filaments as either tight bundles or as homogeneous meshworks [52], [53], we predict that filament remodeling by dynamin2 coordinates the formation of actin filament bundles. Because actin filament crosslinks are important architectural motifs within lamellipodial and lamellar actin networks and for coupling F-actin at sites of cell adhesion [10], [33], a mechanism that defines where, when and how crosslinked filaments are created could contribute to integrating actin networks at the leading edge.

Dynamin2 of the distal lamellipod influenced lamellar actin networks. Formation of nascent actomyosin punctae visualized using GFP-MLC2 was uncontrolled in cells lacking dynamin2. Although the spatiotemporal relationship between GFP-MLC2 punctae and actin filaments were not resolved in our experiments, we suggest that perturbations in nascent actomyosin arise in dynamin2-depleted cells from perturbations in lamellipodial actin filament organization. Recent findings that components of lamellar actomyosin arise, in part, from actin filaments of the lamellipod are consistent with this idea [2], [4]–[6], [54]. The mechanism by which dynamin2 controls actomyosin assembly remains to be determined but it is appealing to speculate that filament remodeling within the lamellipod creates bundled filament templates that are optimal for co-assembly with myosin II.

Finally, lamellar F-actin retrograde flow and the appearance of a distinct border between the lamellipodium and lamellum depended on dynamin2. A hallmark of the lamellipodium-lamellum interface is the transition between fast and slow retrograde F-actin flow [7], [41]. Focal adhesions are thought to trigger the transition from fast to slow flow via interactions with flowing F-actin [6], [7], [9], [43]. α-Actinin is one of several molecular links between flowing F-actin and adhesions [44]–[46]; α-actinin also mediates force transmission to the extracellular matrix [33], [55]–[57]. Adhesion assembly as measured from the rate of GFP-paxillin accumulation was unperturbed in cells lacking dynamin2, however, adhesion width was decreased and accumulation of mCh-α-actinin at new adhesions was delayed. Together with the increased lamellar retrograde flow in cells lacking dynamin2, these observations support the idea that dynamin2 promotes engagement of F-actin at maturing focal adhesions to slow lamellar retrograde flow. Although contractile forces also contribute to adhesion maturation [58]–[60], recent evidence highlights the importance of actin filament organization, particularly filament crosslinking, for adhesion assembly and maturation [9], [10], [33], [61]. We suggest that actin filament remodeling by dynamin2 integrates lamellipodial and lamellar actin networks and their interactions at adhesions.

How does dynamin2 remodel lamellipodial actin filaments and specify filament crosslinking? Emerging biochemical studies of the actions of dynamin2 on actin filaments, together with our cellular experiments, provide some clues. First, dynamins exhibit two actions on actin filaments *in vitro*: filament bundling and severing. Dynamin1 bundled actin filaments *in vitro* [18] and filament bundling by dynamin2 *in vitro* was enhanced by cortactin [21]. Notably, GTP hydrolysis by dynamin2 and cortactin remodeled filaments *in vitro*, creating loosely bundled filaments. Such loose bundles may be ideal templates for crosslinking by α-actinin, an elongated molecule ∼37 nm in length [62], or by myosin II. Dynamin2 and cortactin also severed filaments *in vitro* [21]. Filament severing by dynamin2 GTPase could promote bundling within dense dendritic meshworks by shortening filaments, allowing them to diffuse into positions favorable for crosslinking as isotropic arrays [63]. Whether or not dynamin2 also specifies the orientation of actin filaments within bundles is unknown, however, an ability of dynamin2 to direct formation of filament bundles of defined polarity would impact actomyosin assembly [64].

Recent evidence indicated a role for dynamin2 in regulating signals in lamellipodia that promote actin dynamics and migration [31]. However, our analysis of GFP-actin dynamics in lamellipodia of U2-OS cells using FRAP did not support a role for dynamin2 in regulating filament turnover in U2-OS cells. In addition, at the levels of depletion achieved using siRNAs (∼90%), dyn2-depleted U2-OS cells retained protrusive activity and migrated slightly faster than control cells into cleared areas scraped into cultured monolayers (Fig. S5). We conclude that dynamin2 in U2-OS cells does not influence actin assembly at the leading edge but, rather, acts on select actin filaments within lamellipodial actin networks to integrate formation and dynamics of lamellar networks and promote their engagement at focal adhesions. Additional experiments are needed to determine how these actin-related activities of dynamin2 influence cell migration. Nevertheless, the long-range consequences of lamellipodial dynamin2 on the global actin cytoskeleton of single cells reported here indicate that its contributions to migration are likely complex. Other evidence implicates dynamin2 in regulating centrosome cohesion [14] and microtubule dynamics [13], which could also influence cell polarity and direction or persistence of migration. Dynamin2 may also orchestrate actomyosin and its linkage at sites of cell-cell adhesion [20] to influence collective cell movements or tissue dynamics. We speculate that dynamin2-dependent actin filament remodeling tunes the cell migration machinery to direct cell movement within different environments and cellular contexts. A general function for dynamin2 in remodeling actin filaments networks could account for the varied effects on the actin cytoskeleton observed when dynamin2 activity was disrupted in different cellular contexts [18], [20]–[23]. Given dynamin's expanding roles in secretion and exocytosis, endocytic recycling and signaling, many of which also involve the actin cytoskeleton, dynamin2 may synergistically remodel actin filaments and cellular membranes as cells execute different cellular processes and behaviors.

## Supporting Information

Figure S1 **Depleting dynamin2 in U2-OS cells does not affect the rate of internalization of integrin β1 or transferrin receptor.** (A) Plotted is the percentage of integrin β1 (upper panel) or transferrin receptor (TfnR) (lower panel) internalized by control and dynamin2-depleted cells vs. time after switch to 37°C. Data are representative of at least three independent experiments. (B) Surface level of integrin β1 (left) and TfnR (right) in control and dyn2-depleted cells. Plotted is the intensity of surface-associated fluorophore-tagged antibody specific for each receptor measured prior to switch to 37°C. (C) Western blots of whole cell lysates prepared from equal numbers of control and dyn2-depleted cells were stained with antibodies to detect dynamin2, integrin β1, TfnR, actin and dynamin1, as indicated; actin was the loading control.
(TIF)

Figure S2 **Dynamin1 is detected at diffraction-limited cytoplasmic punctae in U2-OS cells, but not at the lamellipodia.** Single frames from movies of dyn2-depleted U2OS cells transiently expressing mCh-dynamin1. mCh-dynamin1 remains predominately cytoplasmic and localized to diffraction-limited punctae; it is not enriched at distal lamellipodia.
(TIF)

Figure S3 **Mutant dynamin2-K_5_E_5_ is faintly detected at some AP2-positive structures and exhibits decreased dynamics compared to WT-dynamin2 at punctate structures on the plasma membrane.** (A) Representative images of fixed dynamin2-depleted cells expressing comparable and low levels of GFP-WT-dynamin2, GFP-dyn2-ΔPRD or GFP-dyn2-K_5_E_5_ as indicated (green), and immunolabeled with an antibody to the AP2 clathrin adaptor complex of the plasma membrane (red). (B) Boxed regions in each panel of (A) are shown at higher magnification. Arrowheads (cyan) indicate punctae of GFP-dyn2- K_5_E_5_ that are enriched near AP2-positive punctae. (C) Frames from timelapse sequences (extracted from Movie S5) of dynamin2-depleted U2-OS cells expressing either GFP-WT-dynamin2 (upper panels) or GFP-dyn2- K_5_E_5_ (lower panels). Numbers correspond to both panels and indicate elapsed time in seconds.
(TIF)

Figure S4 **Immunolabeling with anti-cortactin in si-control-treated and si-dynamin2-treated U2-OS cells.** (A) Representative images of control and dyn2-depleted fixed cells immunolabeled with anti-cortactin (red) and Alexa488-phalloidin (cyan-blue). Arrowheads indicate regions along the cell periphery where anti-cortactin immunolabeling is enhanced. (B) Cell lysates from equal numbers of control and dyn2-depleted cells were subjected to electrophoresis in 10% polyacrylamide gels followed by transfer to nitrocellulose for detection of cortactin and Vav1/2. Expression of cortactin or Vav1/2 were not perturbed in dyn2-depleted cells. A cell lysate from Jurkat cells was used as a positive control for the anti-Vav antibody.
(TIF)

Figure S5 **Dynamin2 influences migration of cells from a wounded monolayer.** Closure of a scratch wound induced in a confluent monolayer of control and dynamin2-depleted U2-OS cells. Still images (5 images/wound; 2 wounds/sample) of the wounded area were obtained over 20 hours and the percentage of initial wound area plotted over time. Data are compiled from four independent experiments.
(TIF)

Table S1 Listed are the primary antibodies used in this study, including the commercial or laboratory source of the antibodies and the dilution at which the reagents were used.
(DOCX)

Movie S1 Representative movies of control siRNA-treated (left) and dyn2-siRNA-treated (right) U2-OS cells transiently expressing GFP-myosin light chain 2 (MLC2) (green) and mCh-α-actinin (red). Images were collected at a single focal plane every 10 s using an EM-CCD camera (512×512 pixels); playback is 90X real-time.
(MOV)

Movie S2 Representative movie of a dynamin2-depleted U2-OS cell transiently expressing GFP-WT-dyn2 (green) and mCh-α-actinin (red). Images were collected at a single focal plan every 3 s using an ORCA ER CCD camera; playback is 60X real-time.
(MOV)

Movie S3 Representative movie of a dynamin2-depleted U2-OS cell transiently expressing GFP-dyn2-ΔPRD (green) and mCh-α-actinin (red). Images were collected at a single focal plane every 3 seconds using an ORCA ER CCD camera; playback is 60X real-time.
(MOV)

Movie S4 Representative movie of a dynamin2-depleted U2-OS cell transiently expressing mCh-dyn2-K_5_E_5_ (green) and GFP-α-actinin (red). Note that the individual channels were pseudo-colored to make them consistent with other panels of Fig. 2 in the text. Images were collected at a single focal plane every 5 s using an ORCA ER CCD camera; playback is 60X real-time.
(MOV)

Movie S5 Representative movies of dynamin2-depleted cells expressing either GFP-WT-dyn2 or GFP-dyn2-K_5_E_5_, as indicated. Data were collected at the ventral plasma membrane using total internal reflection fluorescence microscopy every 2 s; playback is 40X real-time.
(MOV)

Movie S6 Representative movies of control- and dyn2-siRNAi-treated U2-OS cells transiently expressing GFP-α-actinin. Images were collected at a single focal plane every 5 s using an ORCA ER CCD camera; playback is 100X real-time.
(MOV)

Movie S7 Representative movies of control (left) and dynamin2-depleted (right) U2-OS cell transiently expressing GFP-paxillin (green) and mCh-α-actinin (red). Images were collected at a single focal plane every 10 s using an EM-CCD camera (512×512 pixel); playback is presented at 190X real-time.
(MOV)

Movie S8 Representative movies control, dyn2-depleted and dyn2-depleted cells expressing mutant dynamin2 proteins as indicated together with GFP-MLC2. Mutant dynamin2 proteins were tagged with mCherry to identify expressing cells (not shown). For each movie, an image stack (5–7 images, 0.4 μm spacing) was collected every 10 s using an EM-CCD camera (512×512 pixel); playback is 190X real-time.
(MOV)

Movie S9 Representative movies of dynamin2-depleted U2-OS cells transiently expressing GFP-dyn2-T141A (green, left) or GFP-dyn2-S61D (green, right) together with mCh-α-actinin (red). Images were collected at a single focal plane every 3 s using an ORCA ER CCD camera; playback is 60X real-time.
(MOV)
