# Supplementary figures and images for: Dynamin2 Organizes Lamellipodial Actin Networks to Orchestrate Lamellar Actomyosin
Source: PLoS One. 2014 Apr 7;9(4):e94330. doi: 10.1371/journal.pone.0094330 (PMC3978067; doi:10.1371/journal.pone.0094330)

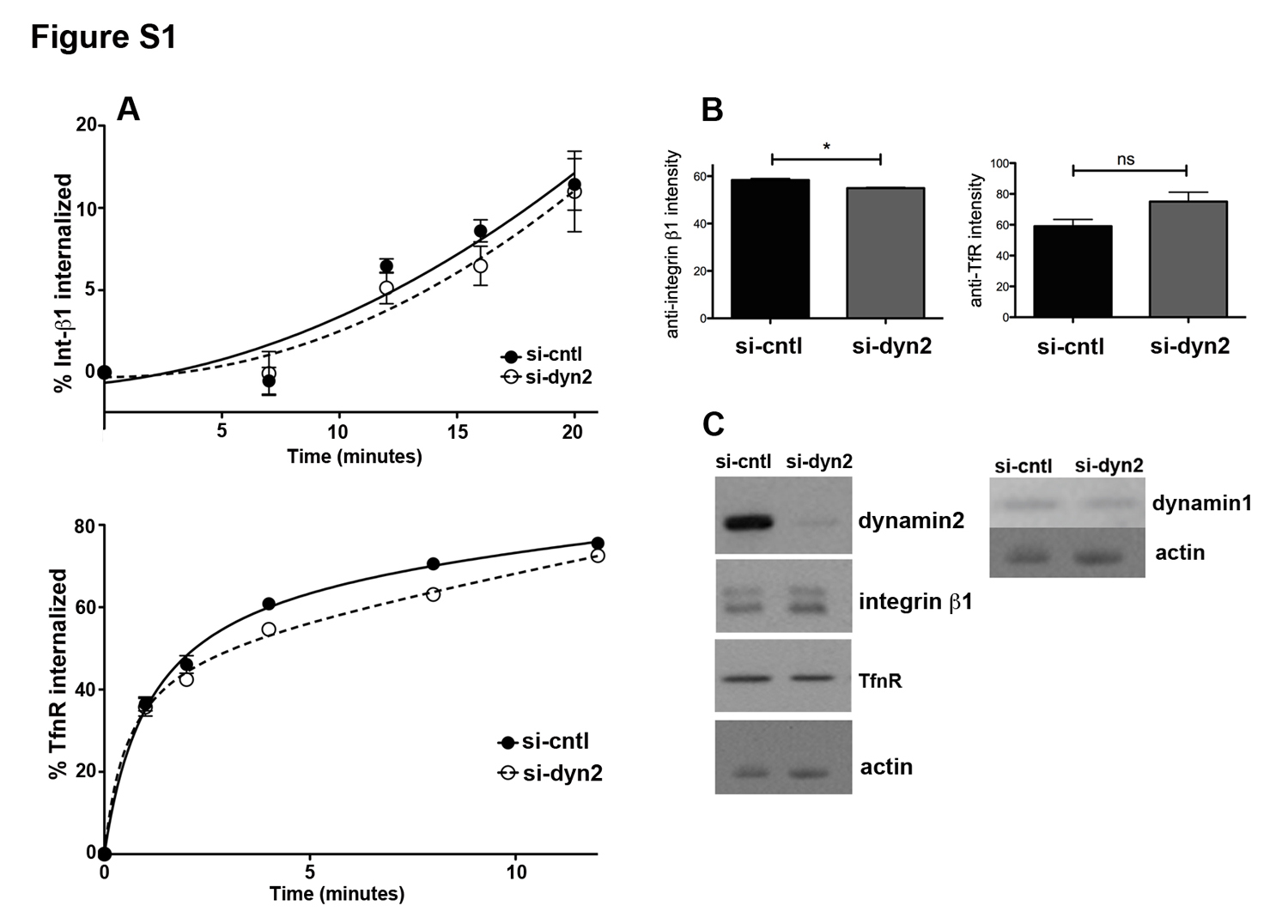

Supplement: Figure S1 — Depleting dynamin2 in U2-OS cells does not affect the rate of internalization of integrin β1 or transferrin receptor. (A) Plotted is the percentage of integrin β1 (upper panel) or transferrin receptor (TfnR) (lower panel) internalized by control and dynamin2-depleted cells vs. time after switch to 37°C. Data are representative of at least three independent experiments. (B) Surface level of integrin β1 (left) and TfnR (right) in control and dyn2-depleted cells. Plotted is the intensity of surface-associated fluorophore-tagged antibody specific for each receptor measured prior to switch to 37°C. (C) Western blots of whole cell lysates prepared from equal numbers of control and dyn2-depleted cells were stained with antibodies to detect dynamin2, integrin β1, TfnR, actin and dynamin1, as indicated; actin was the loading control. (TIF) [file pone.0094330.s001.tif]

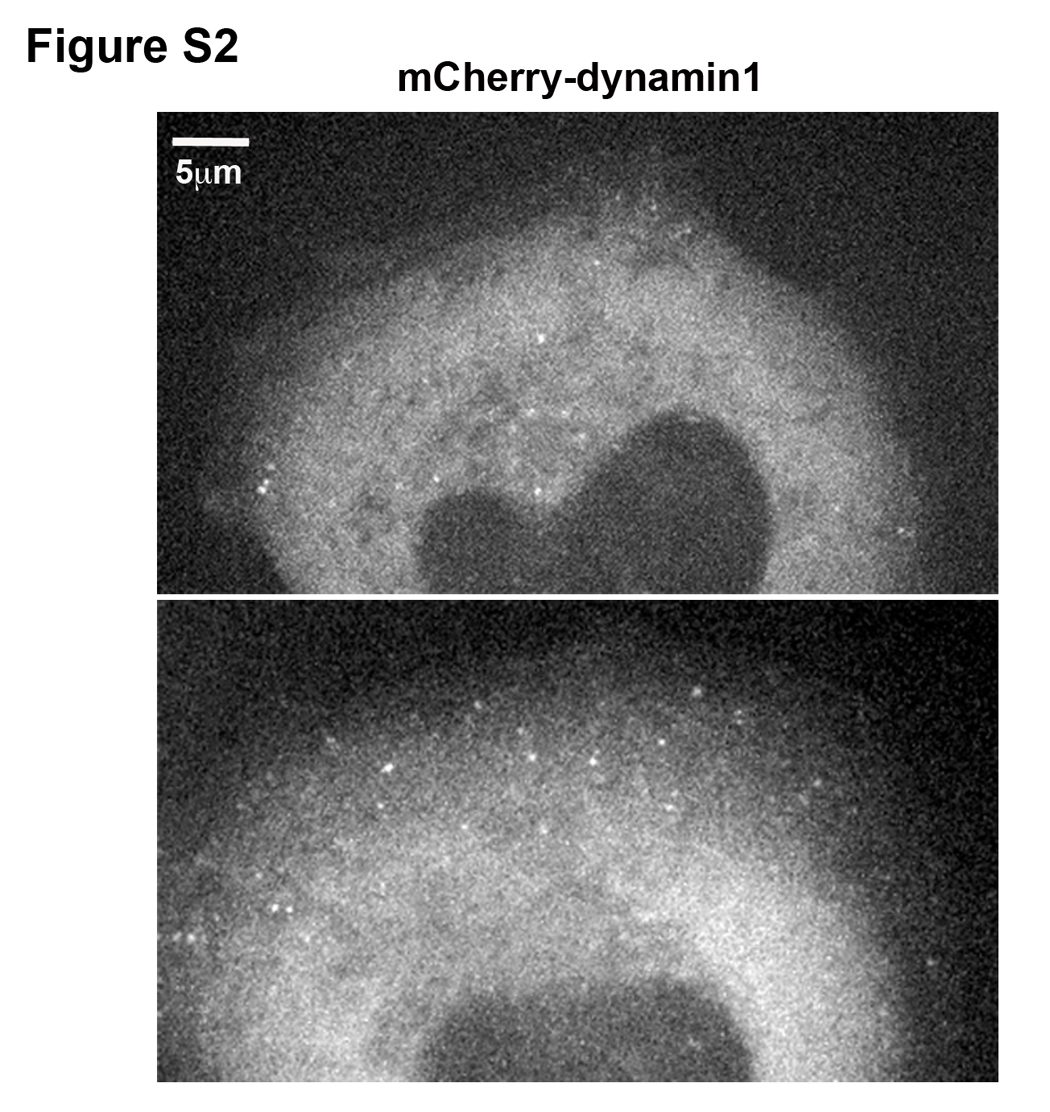

Supplement: Figure S2 — Dynamin1 is detected at diffraction-limited cytoplasmic punctae in U2-OS cells, but not at the lamellipodia. Single frames from movies of dyn2-depleted U2OS cells transiently expressing mCh-dynamin1. mCh-dynamin1 remains predominately cytoplasmic and localized to diffraction-limited punctae; it is not enriched at distal lamellipodia. (TIF) [file pone.0094330.s002.tif]

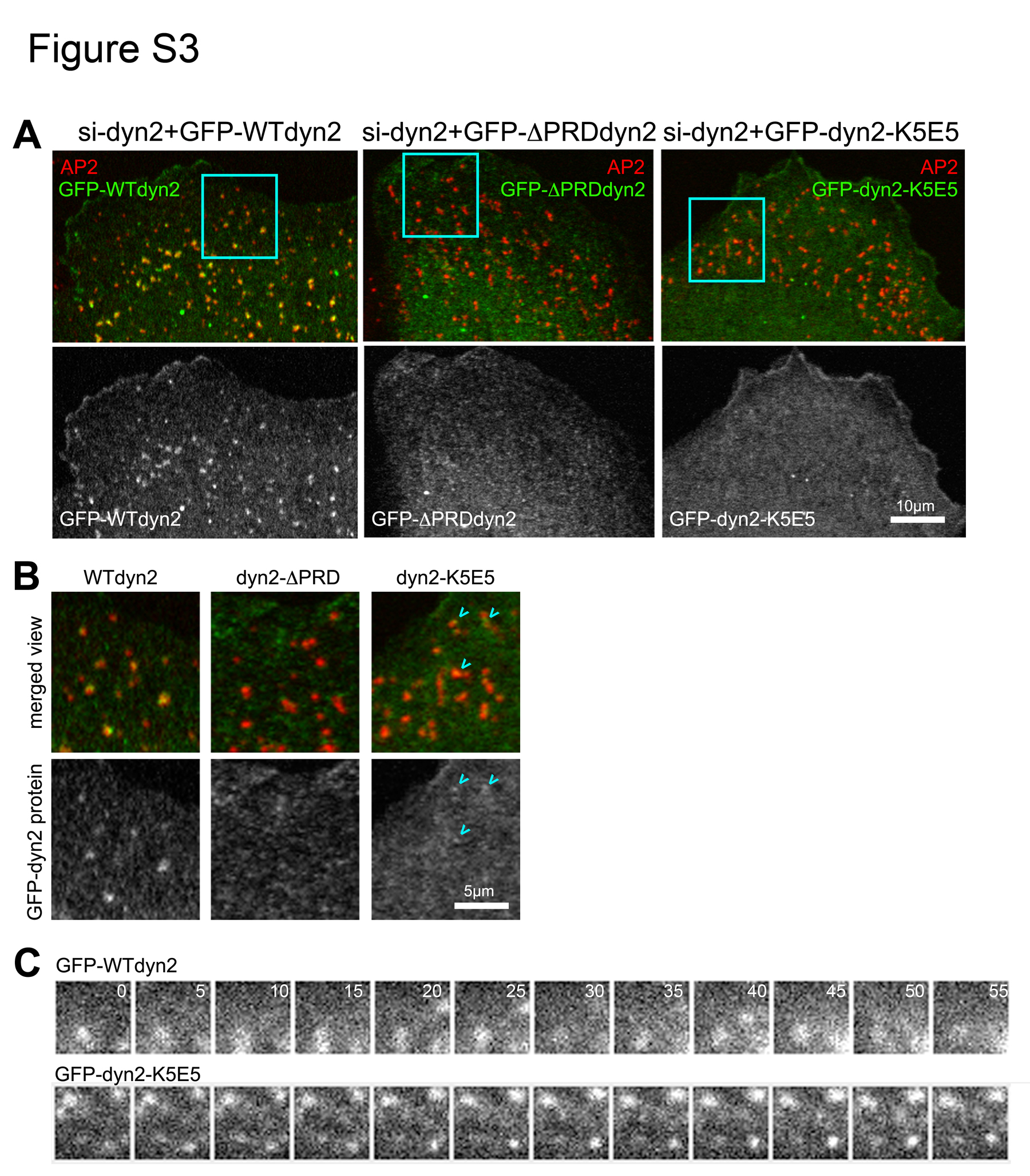

Supplement: Figure S3 — Mutant dynamin2-K5E5 is faintly detected at some AP2-positive structures and exhibits decreased dynamics compared to WT-dynamin2 at punctate structures on the plasma membrane. (A) Representative images of fixed dynamin2-depleted cells expressing comparable and low levels of GFP-WT-dynamin2, GFP-dyn2-ΔPRD or GFP-dyn2-K5E5 as indicated (green), and immunolabeled with an antibody to the AP2 clathrin adaptor complex of the plasma membrane (red). (B) Boxed regions in each panel of (A) are shown at higher magnification. Arrowheads (cyan) indicate punctae of GFP-dyn2- K5E5 that are enriched near AP2-positive punctae. (C) Frames from timelapse sequences (extracted from Movie S5) of dynamin2-depleted U2-OS cells expressing either GFP-WT-dynamin2 (upper panels) or GFP-dyn2- K5E5 (lower panels). Numbers correspond to both panels and indicate elapsed time in seconds. (TIF) [file pone.0094330.s003.tif]

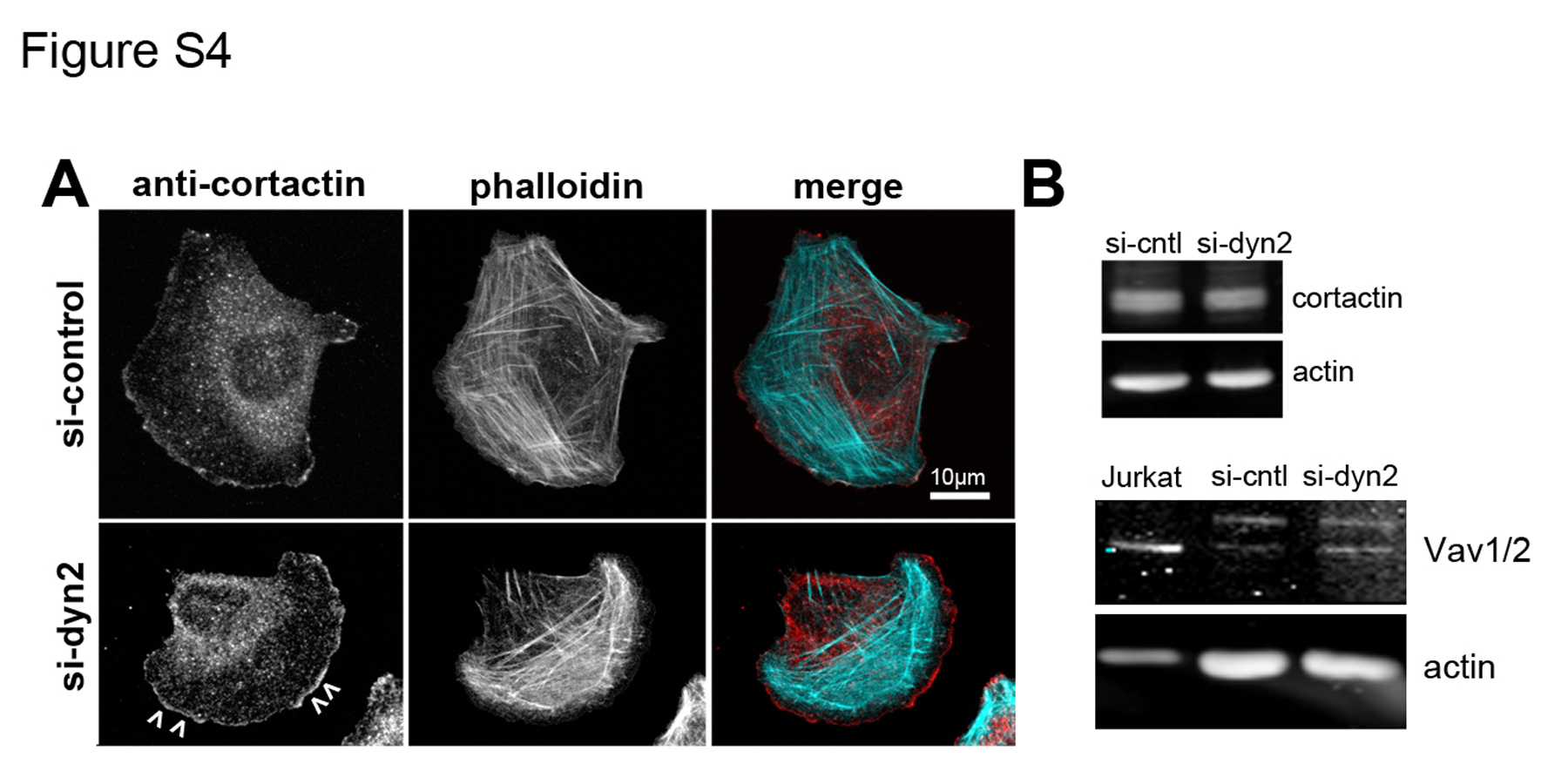

Supplement: Figure S4 — Immunolabeling with anti-cortactin in si-control-treated and si-dynamin2-treated U2-OS cells. (A) Representative images of control and dyn2-depleted fixed cells immunolabeled with anti-cortactin (red) and Alexa488-phalloidin (cyan-blue). Arrowheads indicate regions along the cell periphery where anti-cortactin immunolabeling is enhanced. (B) Cell lysates from equal numbers of control and dyn2-depleted cells were subjected to electrophoresis in 10% polyacrylamide gels followed by transfer to nitrocellulose for detection of cortactin and Vav1/2. Expression of cortactin or Vav1/2 were not perturbed in dyn2-depleted cells. A cell lysate from Jurkat cells was used as a positive control for the anti-Vav antibody. (TIF) [file pone.0094330.s004.tif]

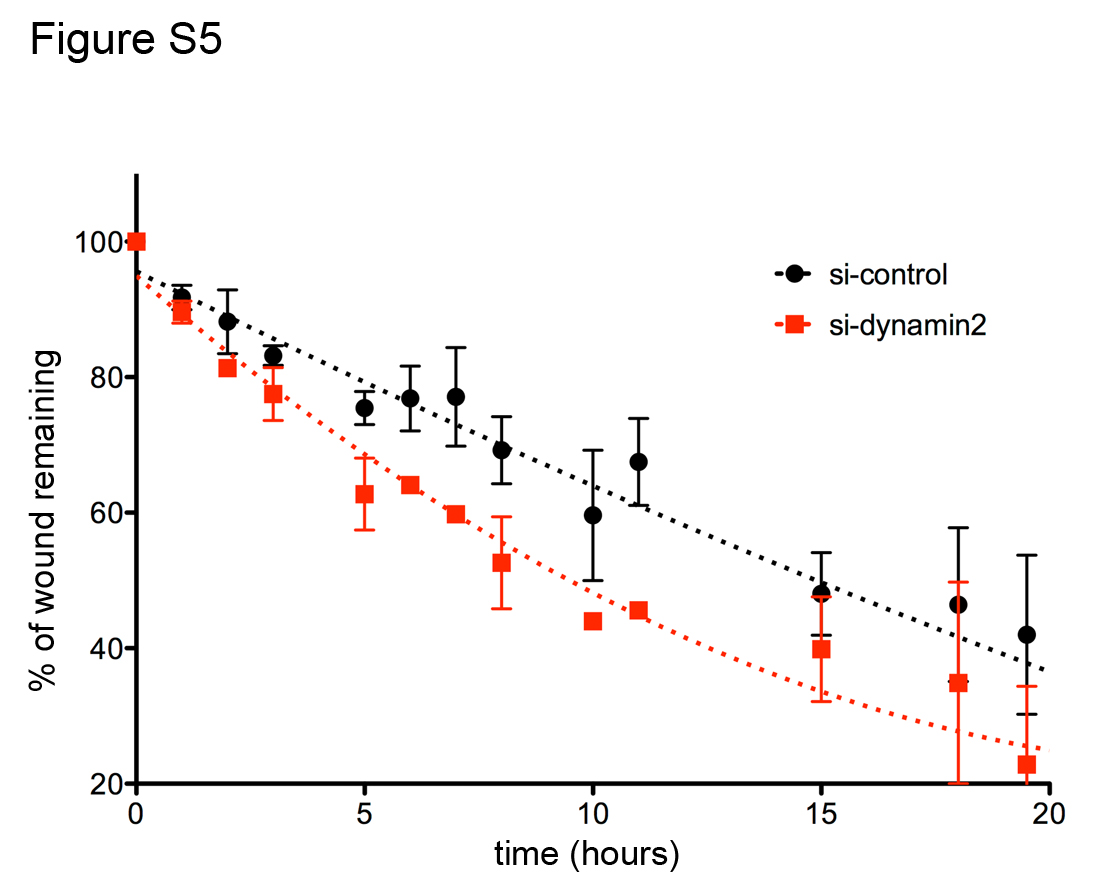

Supplement: Figure S5 — Dynamin2 influences migration of cells from a wounded monolayer. Closure of a scratch wound induced in a confluent monolayer of control and dynamin2-depleted U2-OS cells. Still images (5 images/wound; 2 wounds/sample) of the wounded area were obtained over 20 hours and the percentage of initial wound area plotted over time. Data are compiled from four independent experiments. (TIF) [file pone.0094330.s005.tif]
