# Supplementary material for: Dynamin2 Organizes Lamellipodial Actin Networks to Orchestrate Lamellar Actomyosin
Source: PLoS One. 2014 Apr 7;9(4):e94330. doi: 10.1371/journal.pone.0094330 (PMC3978067; doi:10.1371/journal.pone.0094330)
Supplement: Table S1 — Listed are the primary antibodies used in this study, including the commercial or laboratory source of the antibodies and the dilution at which the reagents were used. (DOCX) [file pone.0094330.s006.docx]

**Table S1. Primary antibodies used in this study**

| **Antibody** | **Source** | **Dilution** |
| --- | --- | --- |
| Goat anti-dynamin2, C-18 | Santa Cruz Biotechnology, Dallas, TX, USA | 1:1000 |
| Goat anti-dynamin1, C-16 | Santa Cruz Biotechnology, Dalls, TX, USA | 1:1000 |
| Mouse anti-actin, C-4 | Millipore, Bilerica, MA, USA | 1:20,000 |
| Rabbit anti-phospho-MLC, T18/S19 | Cell Signaling, Danvers, MA, USA | 1:1000 |
| Rabbit anti-MLC2 | Cell Signaling, Danvers, MA, USA | 1:1000 |
| Mouse CD71 R-PE conjugate (TfR) | Invitrogen, Grand Island, NY, USA | 1:200 |
| Mouse CD29 R-PE conjugate (β1) | Santa Cruz Biotechnology, Dallas, TX, USA | 1:100 |
| Mouse anti-CD71 (TfR) | Santa Cruz Biotechnology, Dallas, TX, USA | 1:1000 |
| Mouse anti-integrin β1 | Gift of James Casanova, Univ. of Virginia | 1:5000 |
| Rabbit anti-phospho ADF/cofilin | Gift of James Bamburg, Colorado State Univ. | 1:2000 |
| Rabbit anti-ADF/cofilin | Gift of James Banburg, Colorado State Univ. | 1:2000 |
| Mouse, anti-cortactin, 4F11 | Upstate Cell Signaling Solutions, NY, USA | 1:5000 |
| Rabbit anti-FAK, C-20 | Santa Cruz Biotechnology, Dallas, TX, USA | 1:1000 |
| Rabbit anti-phospho-FAK | Cell Signaling, Danvers, MA, USA | 1:1000 |
| Rabbit anti-Vav | Cell Signaling, Danvers, MA, USA | 1:1000 |
| Mouse anti-AP2 (clone AP.6) | Gift of James Casanova, Univ. of Virginia | 1:100 |
